# Evaluation of an AI-Based TB AFB Smear Screening System for Laboratory Diagnosis on Routine Practice

**DOI:** 10.3390/s22218497

**Published:** 2022-11-04

**Authors:** Hsiao-Ting Fu, Hui-Zin Tu, Herng-Sheng Lee, Yusen Eason Lin, Che-Wei Lin

**Affiliations:** 1 Division of Laboratory Medicine, Kaohsiung Veterans General Hospital Tainan Branch, Tainan 701, Taiwan; 2 Department of Biomedical Engineering, National Cheng Kung University, Tainan 701, Taiwan; 3 Department of Pathology and Laboratory Medicine, Kaohsiung Veterans General Hospital, Kaohsiung 813, Taiwan; 4 Graduate Institute of Human Resource and Knowledge Management, National Kaohsiung Normal University, Kaohsiung 813, Taiwan; 5 Institute of Gerontology, College of Medicine, National Cheng Kung University, Tainan 701, Taiwan; 6 Medical Device Innovation Center, National Cheng Kung University, Tainan 701, Taiwan; 7 Institute of Medical Informatics, College of Electrical Engineering and Computer Science, National Cheng Kung University, Tainan 701, Taiwan

**Keywords:** acid-fast bacilli, tuberculosis, artificial intelligence

## Abstract

The most robust and economical method for laboratory diagnosis of tuberculosis (TB) is to identify mycobacteria acid-fast bacilli (AFB) under acid-fast staining, despite its disadvantages of low sensitivity and labor intensity. In recent years, artificial intelligence (AI) has been used in TB-smear microscopy to assist medical technologists with routine AFB smear microscopy. In this study, we evaluated the performance of a TB automated system consisting of a microscopic scanner and recognition program powered by artificial intelligence and machine learning. This AI-based system can detect AFB and classify the level from 0 to 4+. A total of 5930 smears were evaluated on the performance of this automatic system in identifying AFB in daily lab practice. At the first stage, 120 images were analyzed per smear, and the accuracy, sensitivity, and specificity were 91.3%, 60.0%, and 95.7%, respectively. In the second stage, 200 images were analyzed per smear, and the accuracy, sensitivity, and specificity were increased to 93.7%, 77.4%, and 96.6%. After removing disqualifying smears caused by poor staining quality and smear preparation, the accuracy, sensitivity, and specificity were improved to 95.2%, 85.7%, and 96.9%, respectively. Furthermore, the automated system recovered 85 positive smears initially identified as negative by manual screening. Our results suggested that the automated TB system could achieve higher sensitivity and laboratory efficiency than manual microscopy under the quality control of smear preparation. Automated TB smear screening systems can serve as a screening tool at the first screen before manual microcopy.

## 1. Introduction

Tuberculosis is an emerging infectious disease worldwide that is treatable, preventable, and curable. However, due to its slow decline, tuberculosis remains a global public health threat. In 2019, about 10 million people fell ill with tuberculosis, and 1.4 million died, more than any other infectious disease [1,2]. Due to the COVID-19 pandemic, there has been a large global drop in the number of people newly diagnosed with TB and an 18% decline, from 7.1 million in 2019 to 5.8 million in 2020, was reported [3]. Sixteen countries accounted for 93% of this reduction, with India, Indonesia, and the Philippines the worst affected. According to the report in 2019, one year before the COVID-19 pandemic, the 30 high TB burden countries accounted for nearly 90% of new TB cases. Eight countries accounted for two-thirds of the total, with India leading the count with 26%, followed by Indonesia (8.5%), China (8.4%), the Philippines (6.0%), Pakistan (5.7%), Nigeria (4.4%), Bangladesh (3.6%), and South Africa (3.6%) [1]. Heads of state and government representatives from all UN members are committed to taking major steps toward building a tuberculosis-free world, including ambitious goals to treat more than 40 million people with tuberculosis and to prevent at least 30 million from becoming ill between 2018 and 2022, through the provision of tuberculosis preventive treatment. According to the data from Taiwan National Infectious Disease Statistics in 2019, there were 3.7 new TB cases and 0.23 deaths per 100,000 people in Taiwan. The tuberculosis treatment success rate of new cases and culture-positive cases were 72.1% and 68.9%, respectively [4].

The most robust and economical method recommended by the World Health Organization (WHO) for the first line of laboratory diagnosis of pulmonary tuberculosis is the acid-fast stain method of sputum smears which relies on manual microscopic examination for acid-fast mycobacteria bacilli (AFB). Although smear microscopy is a low-cost and widely used method, its sensitivity is only 50–60% in pulmonary TB [5]. The fluorescence microscopy is more sensitive than Ziehl–Neelsen stain smear microscopy, but the specificity is similar [6]. The WHO suggested that 1.1 microscopy laboratories should be available for every 100,000 people, and be able to perform quality-assured AFB microscopy [2]. To increase the positive rate, it requires three sputum samples in a row for each potential TB patient (72% at the first samples to 76% with three samples’ examination) [7]. AFB smear microscopy is labor-intensive work, and sensitivity may vary with the experience of examiners, smear quality, eye fatigue, etc. Although some molecular-based methods, such as GeneXpert MTB/RIF can assist in the identification of TB, its high cost means it is unlikely to be affordable in many high TB-burden countries [8]. The gold standard for the diagnosis of tuberculosis is still AFB and the fluorescent microscopic examination of smear or bacteriological confirmation by the culture method. Recently, some automated TB smear microscopy systems have been developed based on artificial intelligence (AI) and big data analysis, which may significantly increase the sensitivity of TB smear microscopy [9,10,11,12,13]. Although all these studies reported better performance than human microscopic examination, most are still in development. Furthermore, this system adopted the idea from digital pathology and used a whole slide scanner to randomly capture the images from the smears. However, the image results are not satisfactory, because TB sputum smears are prepared by hand and the specimens are not smoothly placed onto the glass slide for scanning, unlike the liquid-based cytological smear. An automatic AFB detection system, equipped with a 20× objective lens and an eight-slide loading tray, was applied in daily routine practice for TB sputum smear in our lab. In this study, we evaluated the accuracy of this system compared to traditional manual smear screening in actual practice on routine screening TB smears. We would also like to figure out the key factors that affect the performance when applying the automated TB smear screening system to replace human manual screening. We found the key parameters that effect the accuracy were mostly smear quality and amount of image capture in a constant area. The outcome of an automatic analysis mainly replies on the input images and setting. For achieving the best performance in automation AI-based systems or devices, we think it is necessary to combine the automation of sample preparation and the image recognition algorithm in a whole system.

## 2. Materials and Methods

### 2.1. Study Design

This was a single-center, retrospective, and double-blind study. The study was approved by Kaohsiung Veterans General Hospital Institutional Review Board (KSVGH22-CT4-04). A total of 5930 TB sputum smears were evaluated in this study, collected from the microbiology lab at Kaohsiung General Hospital. The data was collected from December 2018 to January 2020. The evaluation periods were divided into two stages. In the first stage (from December 2018 to March 2019), 1014 smears were analyzed based on the original setting of the machine, which randomly captured 120 images per smear. In the second stage (from April 2019 to January 2020), the image captured field was increased to 200 images per smear, and 3902 smears were analyzed.

### 2.2. Procedures

**Figure 1:**
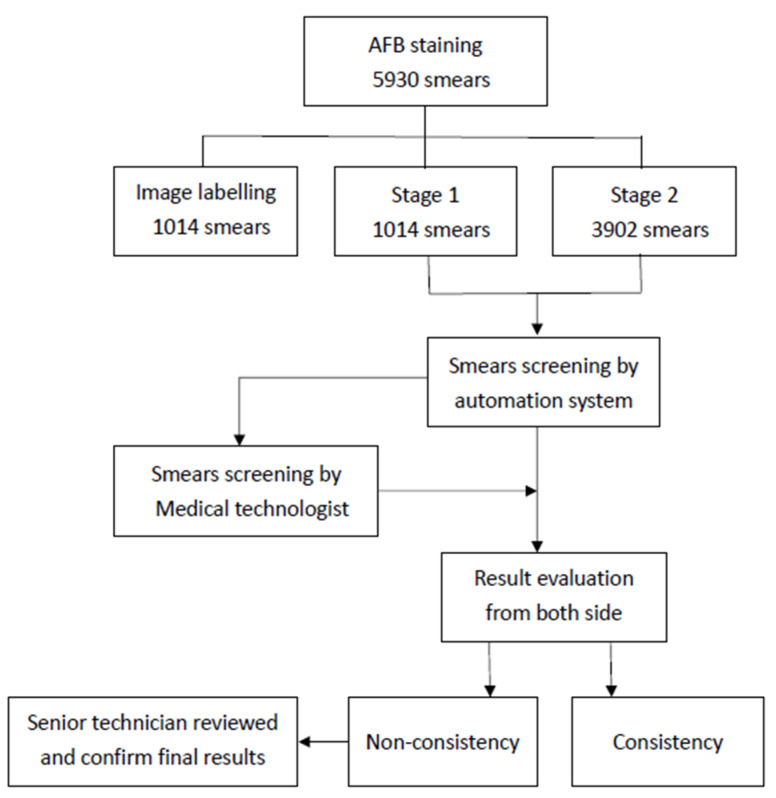
The general workflow of image processing and comparison of performance.

The sample collection, handling, preparation, and AFB smear microscopy were based on the lab guideline authority by the biosafety committee. The sputum samples were spread on a fixed area of 1 cm × 2 cm on the slide and treated with Ziehl–Neelsen stain [14]. All of the slides were loaded into the automated system and scanned by the system. After the system finished the reading, the slides were reviewed by a microbiological medical technologist without knowing the results of the system. Both sides of the results were checked by an independent microbiology medical technologist. The general workflow diagram was illustrated in Figure 1.

### 2.3. AI-Based Automatic TB Detection Device

**Figure 2:**
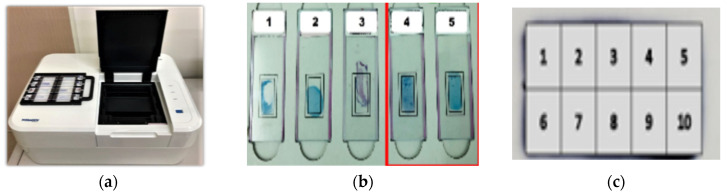
The automated microscopy system: (**a**) an eight-slide tray in one run. (**b**) The fixed 1 cm × 2 cm area where microscopy scanning. The slides of 1, 2 and 3 were inadequate smears, the slides of 4 and 5 (marked with red square) were adequate smears. The number represents the five different slides. (**c**) The fixed 1 cm × 2 cm area on the slide was divided into 10 small areas to be scanned one by one. The 10 areas were marked with the number from 1 to 10. When the suspicious bacteria are detected, the system will indicate the location of area number.

**Figure 3:**
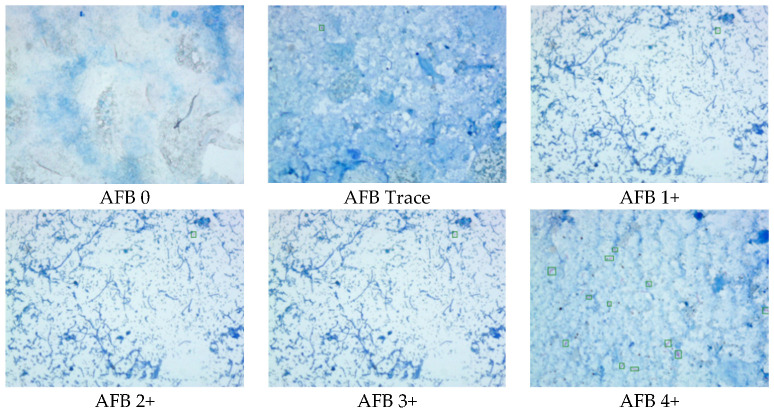
The AFB staining images from trace to 4+.

An automated microscope system (µ-Scan 1.1, Wellgen Medical, Kaohsiung, Taiwan) was used for TB detection [15]. The system consists of two components: hardware and software. The hardware included an eight-slide tray and a microscopic digital camera with an auto-focusing and slide-scanning mechanism to cover the specimen based on WHO recommendations (300 fields @1000× oil lens) (Figure 2a). The system is designed to only scan the 1 cm × 2 cm fixed area and capture the images (Figure 2b). The fixed 1 cm × 2 cm area was divided into ten parts and was scanned one by one (Figure 2c). The software uses an image recognition algorithm to detect and classify positive AFB from level 0 to 4+ (Figure 3). This device can be loaded with eight slides on a tray per run, and each slide takes about 4 min to scan and obtain results. The microscopic images were digitally and randomly captured and stored. In the detection phase, candidate AFBs were marked and differentiated from other substances and tissues in the smear based on color and morphological features. In the classification phase, the feature parameters were extracted from AFB candidates as the input parameters to a proprietary classifier. The results were recorded as positive if any AFB was identified in the image of the slide. Senior medical technologists reviewed all the images and slides to confirm the consistency and evaluate the system’s performance. The training system is a hybrid system including supervised training (targeting bacilli-like objects) and unsupervised training (putting the bacilli-like objective into the convolutional neural network (CNN) for deep learning). The algorithm setting was developed by the manufacturer (µ-Scan 1.1, Wellgen Medica, Kaohsiung, Taiwan). The system applied supervised training to identify the targets of interest based on the morphology (e.g., color, size, length-to-width ratio, etc.) of a TB bacillus. All the targets of interest were processed through a deep learning algorithm for acid-fast bacillus recognition based on the convolutional network work (CNN) framework and other modified methods to fine-tune the algorithm by adjusting the thresholds. Then the certified medical technicians reviewed the results from the algorithm validation as the gold standard, and identified false positive and false negative targets of interest to be re-enrolled into the dataset by the machine learning framework to further improve the algorithm.

### 2.4. Data Interpretation

The evaluation of test performance is based on overall accuracy, sensitivity, and specificity. Statistical analysis was performed using IBM SPSS Statistics, Version 25.0. Armonk, NY, USA.

## 3. Results

### 3.1. The Performance of the Automation System

**Figure 4:**
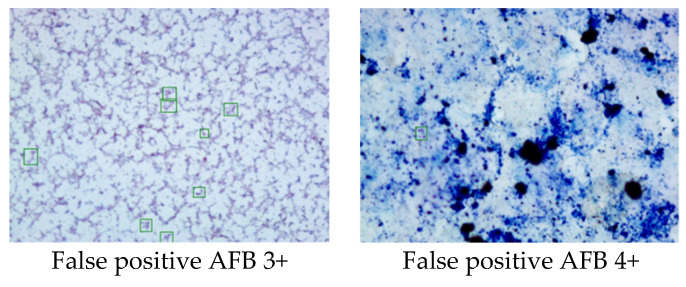
The two false-positive cases due to the poor smear staining quality.

When the study started in December 2018, the first test results (from December 2018 to March 2019, *n* = 1014) were as follows: the sensitivity and specificity were 60% (75/125) and 95.7% (851/889). After a series of imaging training and testing and an increase in the scanning area by 40% (from 120 digital images to 200), the second test results (from April 2019 to January 2020, *n* = 3902) were improved: the sensitivity and specificity were 77.4% (460/594) and 96.6% (3197/3308), respectively (Table 1). During the study, some smears were not as consistent during the sampling process. A total of 709 smears were later excluded from this study due to incomplete stain removal (*n* = 325), smear location shift (*n* = 89), smear being too thick (*n* = 49), smear being too thin (*n* = 110), smear dropping off (*n* = 96), and other reasons due to atypical mycobacterial morphology (*n* = 40) (Table 2, Figure 2b and Figure 4). Thus, the overall results of accuracy, sensitivity, and specificity were 95.2 (3040/3193), 85.6% (406/474), and 95.2% (2634/2719), respectively (Table 1).

**Figure 5:**
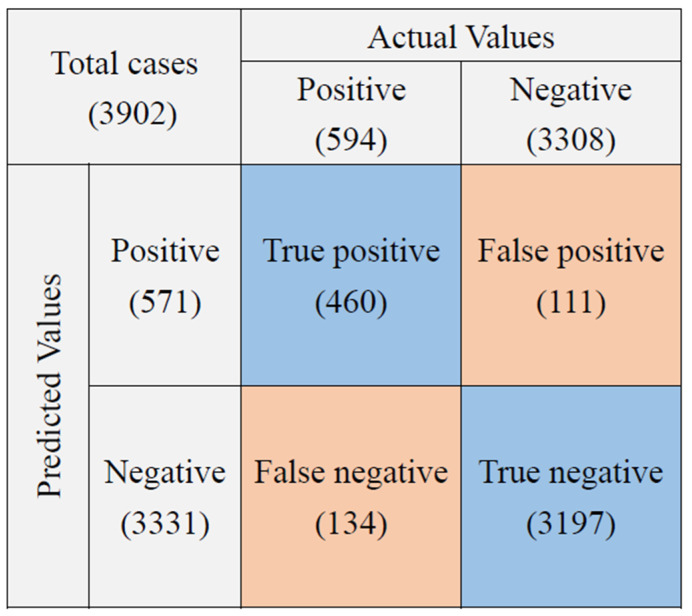
The confusion matrix of automation system with 200 images capture setting.

**Figure 6:**
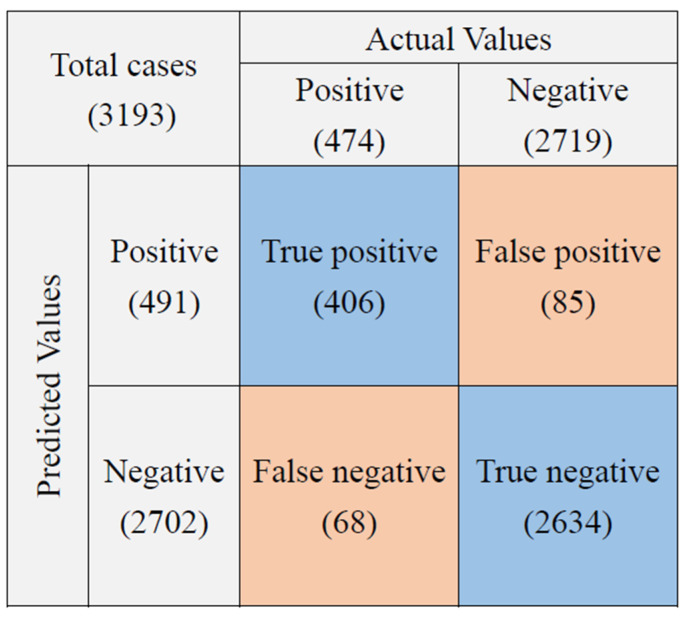
The confusion matrix of automation system with 200 images capture setting after removal of inadequate cases.

We also compared the performance of the automated TB smear microscopy system with manual microscopy performed by medical technologists. Manual microscopy was superior to the automated TB smear microscopy system. The sensitivity, specificity, and accuracy of manual TB smear microscopy were 84.5%, 100%, and 97.6%, respectively, while those of the automated system were 77.4%, 96.6%, and 93.7%, respectively (Table 3, Figure 5). However, after excluding inadequate cases, the sensitivity of the automation system increased to 85.7%, which is higher than manual microscopy with a sensitivity of 82.1% (Table 4 and Figure 6). Furthermore, about 91.4% of cases were reported as positive by both the automation system and technologists, and only <0.1% of cases were missed and reported as negative by both. Overall, the error rate of the automation system (6.32%) was higher than our technologists (2.31%) (Table 5). After excluding the inadequate cases, the error rate of the automation system decreased to 4.73%, while that of technologists slightly increased to 2.6% (Table 6). There were two positive cases missed out by both of them.

### 3.2. The Consistency between the Automated System and Manual Microscopy

In our result, the consistency between the automated system and manual microscopic examination was 100% on AFB results on 2+ to 4+ smears (within ±1 to 2 levels were acceptable). The consistency discrepancy mainly came from smears in trace and 1+ and trace, 39.1%, and 89.1%, respectively (Table 7). This system recovered 85 smears initially found negative from manual microscopic examination (one smear from 2+, 19 smears from 1+, and 65 smears from scanty). However, the automatic system missed 66 smears reported as positive from manual microscopic examination (5 smears from 1+ and 61 smears from scanty) (Table 8).

## 4. Discussion

In the microbiology laboratory, the AFB staining and fluorescence microscopy of sputum smear is still considered standard procedures to confirm Mycobacteria. They are also the most economical, rapid, and readily available methods. However, AFB smear microscopy is labor-intensive work, and the sensitivity may vary with the experience of examiners, smear quality, eye fatigue, etc. Thus, the application of an automatic AI-based system in TB smear examination may provide a constancy of performance and assist medical technologists in carrying out the first screen. We hope such automatic AFB screening system can perform high-throughput screening to increase the positive detection rate.

We applied an automatic TB screening system on AFB smear microscopy in our hospital. In our on-site test, the automated TB scan system achieved an accuracy of 95.2%, sensitivity of 85.7%, and specificity of 96.9%. When comparing the smear microscopy results with culture, the manual microscopy’s overall performance was superior to the automation system. However, after excluding inadequate cases, the sensitivity of the automation system increased to 85.7 from 77.4%, which was higher than manual microscopy with a sensitivity of 82.1%. The error rate of the automation system was 3.7% higher than manual microscopy (2.6%). The design of an image recognition algorithm is to maximize sensitivity, therefore false positives may occur, and those cases need to be ruled out by technologists. All the false positive cases were trace or 1+ levels and accounted for 1.7% in total cases and 11.1% in positive cases detected by the automated system. The automation system showed acceptable consistency with manual microscopy at the higher level, such as 2+, 3+, and 4+. It may save 24.5% of screening time on positive cases for manual microscopy.

In 2012, Lewis et al. reported a smear microscopy system with the sensitivity and specificity of 75.8% and 43.8% [9]. In 2019, Lopez-Garnier et al. reported a CNN automatic TB diagnostic system with high accuracy (96.6%, and a sensitivity and specificity ranging from 91% to 99%. From 2012 to now, numerous AI-based images diagnosis systems have been reported, and the overall performance showed dramatic improvement. Such an improvement in AI development may be due to the progress of algorithms and computing capability. However, we found training images had very different qualities, such as color pixels; this may lead to overfitting outcomes. That may imply that most systems might not be universal. Compared to those studies, our proof-of concept study showed acceptable results. For seeking the best performance, we think the standardized sample preparation and customized algorithm fine-tuning are needed.

Furthermore, we found several issues worth mentioning when applying automated microscope systems in clinical laboratories: (a) the smear location: the manual smear technique needs to be standardized so that the smear location is systematically the same place. If the smear location is not standardized, it may affect the accuracy of the automated system. (b) Stain quality: the manual stain technique also affects the automated system’s performance, since the recognition software uses color as an important parameter for detecting AFB. An automatic smear and stain system, in addition to our automated microscope, may improve the quality of the smears. The main purpose of developing an AI-based TB automatic screening system is to save the technician’s workload and focus on the suspected positive slide of AFB. We consider such AI-based TB automation system as an assistance tool, instead of a replacement for the diagnosis from medical technologists. This system can facilitate the junior technicians to minimize error rate, due to the lack of experience.

Through advances in artificial intelligence (AI), machine learning (ML), and deep learning technology (DL), medicine is shifting into the digital medicine era. Computer technology not only can be used to make medical records, transport data, or in analysis, but also be expected to function with AI to diagnose diseases, make medical decisions, give health suggestions, and even treat patients [16]. AI has been massively applied in the development of medical devices and software. In the medical laboratory, most works still rely on pathologists, cytopathologists, and medical technologists, and manual operation remains. With a large number of samples needing to be handled in medical laboratories every day, the role of AI in laboratory medicine is expected to improve the accuracy of detection and laboratory workflow, help avoid diagnosis errors, and increase efficiency. Based on a survey, about 15.6% of organizations currently use AI in lab diagnosis. However, most still remain uncertain about adopting AI in medical diagnosis. One concern is the lack of proven clinical benefits. Therefore, our lab-side routine practice data showed promising results and support and ensured the reliability of AI-based medical devices’ performance [17]. The proof-of-concept of this AI-based medical devices at our lab can provide reassurance to other laboratories when they were considering the installation of such expensive automation systems or devices.

This TB smear automatic screening system is rapid and easy to operate for lab staff. The light and neat device design can easily settle on the table in any laboratory space. A good medical device design should consider the applicability and convenience of the potential users. The standard AFB staining for TB diagnosis only requires simple sputum smear preparation. With the help of automatic TB bacteria detection systems, the capacity of AFB tests per year can be increased. Automatic TB bacteria screening systems equipped with AI should be installed in high tuberculosis burden countries with limited medical resources, especially in developing countries or remote areas.

Besides bacteria morphology, gene identification, chest X-ray images, and biosensor medical devices, such as electronic noses, can detect tuberculosis via patient’s breath and even culture samples of Mycobacterium tuberculosis. The odor of bacteria itself or patients with TB infection can be analyzed via e-nose [18]. These need machine learning and deep learning to set up algorithms to work on the device. In the future, AI can help develop needed tools for TB diagnosis and increase the accuracy of current TB diagnosis methods.

## 5. Conclusions

In conclusion, the automated TB microscopy system can achieve the same high performance as manual microscopy. We found that the accuracy of this system was mainly influenced by the scanning area and the smear staining quality. By increasing the number of captured images on each slide, the positive rate of TB screening will also be increased. To achieve better staining quality, the establishment of standardized staining procedures is essential. It could assist medical technologists in screening positive cases, which may be missed by manual microscopy screening. The standardized smear preparation protocol is needed for the adoption of the automation system. A regularly updating algorithm from clinical data for training, validation, and testing may improve the accuracy.

## Figures and Tables

**Table 1:** The performance of the automated TB smear microscopy system.

	Stage 1	Stage 2	Included Cases with Adequate Smear
Period	19 December 2018– 28 March 2019	29 March 2019– 31 December 2019	
True positive	75	460	406
True negative	851	3197	2634
False positive	50	111	85
False negative	38	134	68
Total cases	1014	3902	3193
Sensitivity	60.0%	77.4%	85.7%
Specificity	91.3%	96.6%	96.9%
Accuracy	95.7%	93.7%	95.2%

**Table 2:** The factors that influence the smear microscopy.

	No. of Cases	Percentage (%)
Incomplete stain	325	45.9%
Smear too thin	110	15.5%
Smear dropped off	96	13.5%
Smear location shift	89	12.6%
Smear too thick	49	6.9%
Atypical TB shape	40	5.6%
Total	709	100%

**Table 3:** The performance of automated TB smear microscopy system and manual microscopy.

	Automated System	Manual Microscopy
True positive	460	502
True negative	3197	3308
False positive	111	0
False negative	134	92
Total cases	3902	3902
Sensitivity	77.4%	84.5%
Specificity	96.6%	100%
Accuracy	93.7%	97.6%

**Table 4:** The performance of the automated TB smear microscopy system and manual microscopy after removal of inadequate cases.

	Automated System	Manual Microscopy
True positive	406	389
True negative	2634	2719
False positive	85	0
False negative	68	85
Total cases	3193	3193
Sensitivity	85.7%	82.1%
Specificity	96.9%	100%
Accuracy	95.2%	97.3%

**Table 5:** The precision and error rate of the automation system and manual microscopy. The “True” represents the cases diagnosed correctly; The “False” represents the cases of wrong diagnosis.

FinTotal Case No.	3902	Manual Microscopy	
		True	False
Automated system	True	3567 91.41%	90 2.31%
	False	243 6.32%	2 0.06%

**Table 6:** The precision and error rate of the automation system and manual microscopy. The “True” represents the cases diagnosed correctly; the “False” represents the cases of wrong diagnosis, after removal of inadequate cases.

Total Case	3193	Manual Microscopy	
		True	False
Automated system	True	2957 92.61%	83 2.60%
	False	151 4.73%	2 0.06%

**Table 7:** The consistency between the automated TB smear microscopy system and manual microscopic examination.

	Manual Microscopy	Automated System	Consistency (%)
Trace	192	75	39.1
AFB 1+	137	122	89.1
AFB 2+	89	89	100.0
AFB 3+	47	47	100.0
AFB 4+	37	37	100.0
Not Found	3308	3308	100.0

**Table 8:** The number of missed cases by the automation system and manual microscopy.

	Missed by System	Missed by Manual Examination
Trace	61	65
1+	5	19
2+	0	1
Total	66	85

## Data Availability

Not applicable.

## References

[1] World Health Organization (2020). Global Tuberculosis Report 2020.

[2] Reid M.J.A., Arinaminpathy N., Bloom A., Bloom B.R., Boehme C., Chaisson R., Chin D.P., Churchyard G., Cox H., Ditiu L. (2019). Building a tuberculosis-free world: The Lancet Commission on tuberculosis. Lancet.

[3] World Health Organization (2021). Global Tuberculosis Report 2021.

[4] Taiwan CDC (2019). Taiwan Tuberculosis Control Repor 2019.

[5] Campelo T.A., Cardoso de Sousa P.R., Nogueira L.L., Frota C.C., Zuquim Antas P.R. (2021). Revisiting the methods for detecting Mycobacterium tuberculosis: What has the new millennium brought thus far?. Access Microbiol..

[6] Steingart K.R., Henry M., Ng V., Hopewell P.C., Ramsay A., Cunningham J., Urbanczik R., Perkins M., Aziz M.A., Pai M. (2006). Fluorescence versus conventional sputum smear microscopy for tuberculosis: A systematic review. Lancet Infect. Dis..

[7] Islam M.R., Khatun R., Uddin M.K., Khan M.S., Rahman M.T., Ahmed T., Banu S. (2013). Yield of two consecutive sputum specimens for the effective diagnosis of pulmonary tuberculosis. PLoS ONE.

[8] Ngabonziza J.C., Ssengooba W., Mutua F., Torrea G., Dushime A., Gasana M., Andre E., Uwamungu S., Nyaruhirira A.U., Mwaengo D. (2016). Diagnostic performance of smear microscopy and incremental yield of Xpert in detection of pulmonary tuberculosis in Rwanda. BMC Infect. Dis..

[9] Lewis J.J., Chihota V.N., van der Meulen M., Fourie P.B., Fielding K.L., Grant A.D., Dorman S.E., Churchyard G.J. (2012). “Proof-of-concept” evaluation of an automated sputum smear microscopy system for tuberculosis diagnosis. PLoS ONE.

[10] Lopez-Garnier S., Sheen P., Zimic M. (2019). Automatic diagnostics of tuberculosis using convolutional neural networks analysis of MODS digital images. PLoS ONE.

[11] Panicker R.O., Soman B., Saini G., Rajan J. (2016). A Review of Automatic Methods Based on Image Processing Techniques for Tuberculosis Detection from Microscopic Sputum Smear Images. J. Med. Syst..

[12] Zingue D., Weber P., Soltani F., Raoult D., Drancourt M. (2018). Automatic microscopic detection of mycobacteria in sputum: A proof-of-concept. Sci. Rep..

[13] Xiong Y., Ba X., Hou A., Zhang K., Chen L., Li T. (2018). Automatic detection of mycobacterium tuberculosis using artificial intelligence. J. Thorac. Dis..

[14] Taiwan Society of Laboratory Medicine (2018). Concentred Smear Acid-Fast Stain Operation Guideline.

[15] Smart Medical Microscope, Wellgen Medical Co., Ltd. https://www.wellgen.info/en-pas.

[16] US Food and Drug Administration (2020). What is Digital Health?.

[17] Paranjape K., Schinkel M., Hammer R.D., Schouten B., Nannan Panday R.S., Elbers P.W.G., Kramer M.H.H., Nanayakkara P. (2021). The Value of Artificial Intelligence in Laboratory Medicine. Am. J. Clin. Pathol..

[18] Rudnitskaya A., Marini F., Tonacci A., Scafile A., Billeci L., Sansone F. (2022). Electronic Nose and Tongue for Assessing Human Microbiota. Chemosensors.

